# Budget Impact of Publicly Reimbursed Prescription Medicines in the Republic of Srpska

**DOI:** 10.3389/fpubh.2015.00213

**Published:** 2015-09-10

**Authors:** Tamara Petrusic, Mihajlo Jakovljevic

**Affiliations:** ^1^Inpharm Co. d.o.o., Banja Luka, Bosnia and Herzegovina; ^2^Faculty of Medical Sciences, University of Kragujevac, Kragujevac, Serbia

**Keywords:** prescription medicines, pharmaceuticals, reimbursement, Republic of Srpska, Bosnia, cost, budget impact, trend

## Health Care Funding and Provision of the Republic of Srpska

Health Care Services in the Republic of Srpska are provided through the primary, secondary, and tertiary care levels. Primary medical services provision happens throughout the network of 53 primary health care facilities and 1 family medicine polyclinic (1). There are approximately 500 pharmacies (2). Secondary and tertiary health care services are provided in two university hospitals, nine regional hospitals, three health centers, two specialized psychiatric clinics, and two specialized medical rehabilitation facilities (3). Insured citizens use health care services in public health care institutions and private institutions who have signed the contract with the Health Insurance Fund. Health care financing is mostly provided by the Health Insurance Fund, out of the mandatory taxes imposed to the employers and employees alike. Coverage for unemployed population is extended from the employed family member premiums (4). Fund is a legal entity established and owned by the State (5). The Ministry of Health and Social Welfare conducts the supervision of the Fund (6). One of the main functions of the Health Insurance Fund is collection of contributions for health insurance and contracting of health services. Complementary direct payment of contributions is made to the Pension and Disability Insurance Fund (7). The health care system in the Republic of Srpska is centralized with the overall power trusted to the Ministry of Health and Social Welfare, the Institute of Public Health, and the Health Insurance Fund. Employee pool in the health care system of the Republic of Srpska consists of approximately 2,400 clinical physicians, 200 dentists, 100 pharmacists, 6,500 nursing staff, and 4,000 administrative and technical staff (1). Gross National Income per capita increased from 8,770 to 9,820 US$ during 2009–2013 span. Total expenditure on health, as a percentage of gross domestic product, was 9.9% in 2012 (last official release) (8). The Republic of Srpska has around a population of 1,327,000 (9), and health expenditure per capita in 2009 amounted to €389 while in 2013 it reached €397 (10). Private and public expenditure on health care has increased in total from 2009 to 2012, and the public share of spending grew from 68.81 to 70.33% (11).

## Official Records on Prescription Medicines

The Agency for Medicinal Products and Medical Devices of Bosnia and Herzegovina issues annual reports on utilization of pharmaceuticals nationwide. These annual reports are commercially available since 2009 (12). This source contains data on medicines prescription, dispensing, and sales provided directly by the pharmaceutical multinationals and domestic manufacturers. Wholesalers are required to provide data on the number of packages of drugs that are imported and wholesale price. This price usually consists of manufacturer’s price plus customs, incremental costs, and the wholesale markup. Domestic producers were required to provide data on the number of packages of drugs that are produced and released into the market with wholesale price (manufacturer’s price plus the wholesale markup). Costs are expressed in Bosnia and Herzegovina’s local currency – convertible mark (BAM). Data are ordered in ATC (Anatomical–Therapeutical–Chemical) codes on first, second, third, fourth, and fifth level of classification (13). Latest report has been made in 2014 for the year 2013. Total of 39 wholesalers provided data. The ratio of domestic and foreign manufacturers of medicines in the total turnover of pharmaceuticals in 2013 was 17 vs. 83% (14). Population health official estimate is being issued annually by the Public Health Institute of the Republic of Srpska (15). It provides us with exact data with regard to health facilities, staffing, and the structure of spending (public and private expenditure). Third source of data supporting these claims was the Agency for Statistics’ publications on gross domestic product and National Health Accounts Statistics for the period from 2009 to 2013 (16).

## Ongoing Pharmaceutical Market Transformation 2009–2013

Observing 5-year time horizon (2009–2013), we see that moderate growth of pharmaceutical market took place. Some ATC code groups recorded far more substantial market changes than others. Thus, systemic hormonal preparations, excluding sex hormones, and insulin value-based turnover increased for 92%, and antiparasitic products, insecticides, and repellents spending decreased for 63%. Changes were also significant in the indication field of blood and blood-forming organs reporting 36% growth, as well as antineoplastic and immunomodulating agents, sensory organs, respiratory system, and alimentary tract and metabolism drugs which increased their sales volume around 30% each. Top 10 ATC second-level code groups in 2009 and 2013 have been renin–angiotensin system agents, antibacterials for systemic use, antineoplastic agents, psycholeptics, drugs used in diabetes, drugs for acid-related disorders, analgesics, calcium channel blockers, anti-inflammatory and antirheumatic products, beta blocking agent, and antithrombotic agents. Consumption of preparations for wounds and ulcers treatment has increased three times. Nasal preparations, digestives, including enzymes, pituitary and hypothalamic hormones, and prescribing antigout preparations have also increased twofold (Table 1).

**Table 1:** **Top 10 ATC second- and third-level drug code groups ranked in 2009 and 2013 according to value-based turnover of dispensed medicines and its growth ratio 2013/2009^a^**.

Top 10 ATC second-level code groups in 2009and 2013 (hierarchy from 2009 appliedin descending order of appearance)	Value of prescriptions dispensed in 2009 (million €)	Value of prescriptions dispensed in 2013 (million €)	Growth ratio 2013/2009	Total increase 2009–2013 (million €)
C09 agents acting on the renin–angiotensin system	25.9	26.8	1.03	0.9
J01 antibacterials for systemic use	21.8	16.2	0.74	5.6
L01 antineoplastic agents	16.1	21.4	1.33	5.3
N05 psycholeptics	13	10.1	0.78	2.9
A10 drugs used in diabetes	12.5	23.1	1.85	10.6
A02 drugs for acid-related disorders	10.5	10.3	0.98	0.2
N02 analgesics	9.6	9.1	0.95	0.5
C08 calcium channel blockers	7.4	5.4	0.73	2
M01 anti-inflammatory and antirheumatic products	7.4	8.9	1.21	1.5
C07 beta blocking agent	6.9	8.1	1.18	1.2
B01 antithrombotic agents	6.8	10.6	1.56	3.8

**Top 10 ATC third-level code groups according to 2013/2009 growth ratio**	**Value of prescriptions dispensed in 2009 (thousand €)**	**Value of prescriptions dispensed in 2013 (thousand €)**	**Growth ratio 2013/2009**	**Total increase 2009–2013 (thousand €)**

C01C cardiac stimulants excluding cardiac glycosides	7.6	122	16.07	114.5
S03A anti-infectives	3.8	36	9.47	32.2
M03A muscle relaxants, peripherally acting agents	37.1	347.6	9.37	310.5
C09D angiotensin II antagonists, combinations	511	2993.1	5.86	2482.1
S01B Anti-inflammatory agents	17.8	99.5	5.59	81.7
R01B nasal decongestants for systemic use	484.6	2350.1	4.85	1865.5
D03A cicatrizants	43.3	169	3.90	125.7
L01A alkylating agents	238.6	876.8	3.67	638.2
V03A all other therapeutic products	53	189.2	3.57	136.2
G02B contraceptives for topical use	19.9	70.5	3.54	50.6

*^a^Latest Republic of Srpska’s official release available [publicly reimbursed prescription medicines only; over-the-counter (OTC) and out-of-pocket citizen payments excluded]*.

Differences in certain ATC drug group consumption are reflecting the changes that have been happening in the national health system. Prices have been reduced, especially for generic drugs where patent protection has expired. A lot of effort has been invested in the implementation of good clinical practice guidelines. That is the reason for reducing antibiotics consumption outsourcing from decreased frequency of nosocomial infections and overprescribing by general practice physicians. Development of novel targeted antineoplastic medicines, primarily the biologicals, as well as increased incidence of malignant diseases influenced constant growth of utilization. The level of obesity is increasing and so is antidiabetics consumption as well as pharmaceuticals indicated in cardiovascular and associated diseases.

## Forecasts of Future Changes

Although there are some promising developments, local pharmaceutical market structure and dynamics is still far from the one responsive to population needs. In the country still recovering from unstable political situation over the last few decades, a lot of progress has been made. The Health Insurance Fund is consistently negotiating with pharmaceutical companies imposing price caps and generic substitution wherever possible. Thus, Fund created more room for innovative therapies reimbursement. That is why authorities are considering marketing approvals of novel drugs indicated in hepatitis, diabetes, targeted antineoplastic agents (17), as well as immunotherapy, which we expect to be available to the patients over the next 5–10 years.

Western Balkan countries share post-socialist legacy of former Yugoslavia in health care management and financing patterns (18). Quite similar but faster development in a large-scale population could be observed in neighboring Serbia (19). This country suffered as well from seriously constrained resource allocation for medicines during global economic recession (20). Signs of early recovery are now clearly present particularly in the oncology indication field (21). Accessibility and affordability of innovative therapies gradually became reality for even the poorest citizens (22).

The Republic of Srpska due to its smaller population size might have more convenient opportunity for sustainable health policy solutions learning from painful lessons of other South-East European transitional health reforms (23). Current developments nationwide present a promise of more responsive pharmaceutical market. The country is likely to be capable of providing more affordable, mostly generic medicines in the upcoming years.

## Conflict of Interest Statement

The authors declare that the research was conducted in the absence of any commercial or financial relationships that could be construed as a potential conflict of interest.

## References

[1] Analysis of Population Health in Republic of Srpska. Banja Luka: Public Health Institute (2013).

[2] Pharmacy Register (2015). Available from: www.vladars.net/Vlada/Ministarstva/MZSZ/PAO/Documents

[3] Agency for Certification, Accreditation and Health Care Improvement of the Republic of Srpska (2015). Available from: www.askva.org/askva/bolnicki-sektor

[4] Health Insurance Act (2015). Available from: http://www.vladars.net/Vlada/Ministarstva/Documents/Zakon o zdravstvenom osiguranju

[5] National Health Insurance Fund (2015). Available from: www.zdravstvo-srpske.org

[6] Ministry of Health and Social Welfare (2015). Available from: www.vladars.net/vlada/ministries/MHSW

[7] Pension and Disability Insurance Fund (2015). Available from: www. fondpiors.org

[8] WHO Global Health Observatory Data Repository. Bosnia and Herzegovina Statistics Summary. Available from: www.who.int/gho/data/node.country.country-BIH

[9] BIH Agency for Statistics and Entity Statistical Offices. Preliminary Results of the 2013 Census of Population, Households and Dwelling in Bosnia and Herzegovina.

[10] WHO Global Health Expenditure Database. Available from: www.who.int/health-accounts

[11] Agency for Statistics of Bosnia and Herzegovina. National Health Accounts Statistics. Sarajevo: Agency for Statistics of Bosnia and Herzegovina (2014).

[12] The Agency for Medicinal Products and Medical Devices of Bosnia and Herzegovina (2015). Available from: http://www.almbih.gov.ba/_doc/publikacije/Promet2013.pdf

[13] WHO Collaborating Centre for Drug Statistic Methodology. ATC/DDD Index (2015). Available from: http://www.whocc.no/atc_ddd_index/

[14] The Agency for Medicinal Products and Medical Devices of Bosnia and Herzegovina (2014). Available from: www.almbih.gov.ba/_doc/publikacije/Promet2013.pdf

[15] Republic of Srpska Public Health Institute. Available from: www.phi.rs.ba

[16] Agency for Statistics of Bosnia and Herzegovina. Available from: www.bhas.ba

[17] Jakovljevic MB Oncology monoclonal antibodies expenditure trends and reimbursement projections in the emerging Balkan market. Farmeconomia. Health Econ Ther Pathways (2014) 15(1):27–32. 10.7175/fe.v15i1.909

[18] Jakovljevic MB. Resource allocation strategies in Southeastern European health policy. Eur J Health Econ (2013) 14(2):153–9. 10.1007/s10198-012-0439-y 23143312

[19] Jakovljevic M Djordjevic N Jurisevic M Jankovic S. Evolution of Serbian pharmaceutical market alongside socioeconomic transition. Expert Rev Pharmacoecon Outcomes Res (2015) 15(3):521–30. 10.1586/14737167.2015.1003044 25592856

[20] Jakovljevic MB Health expenditure dynamics in Serbia 1995-2012, hospital pharmacology. Int Multidiscip J (2014) 1(3):180–3.

[21] Dagovic A Zugic A Jakovljevic MB Macroeconomic policy impact on oncology-related public expenditure in an emerging European market – signs of early recovery. Ser J Exp Clin Res (2015) 16(1):43–50.

[22] Jakovljevic M Jovanovic M Lesch MO Accessibility and affordability of alcohol dependency medical care in Serbia. Front Psychiatry (2015) 5:192 10.3389/fpsyt.2014.00192 25628574 PMC4290475

[23] Jakovljevic M Vukovic M Chia-Ching C Antunovic M Dragojevic-Simic V Velickovic-Radovanovic R Do health reforms impact cost consciousness of health care professionals? Results from a nation-wide survey in the Balkans. Balkan Med J (2015). 10.5152/balkanmedj.2015.15869 PMC4767315 26966613

